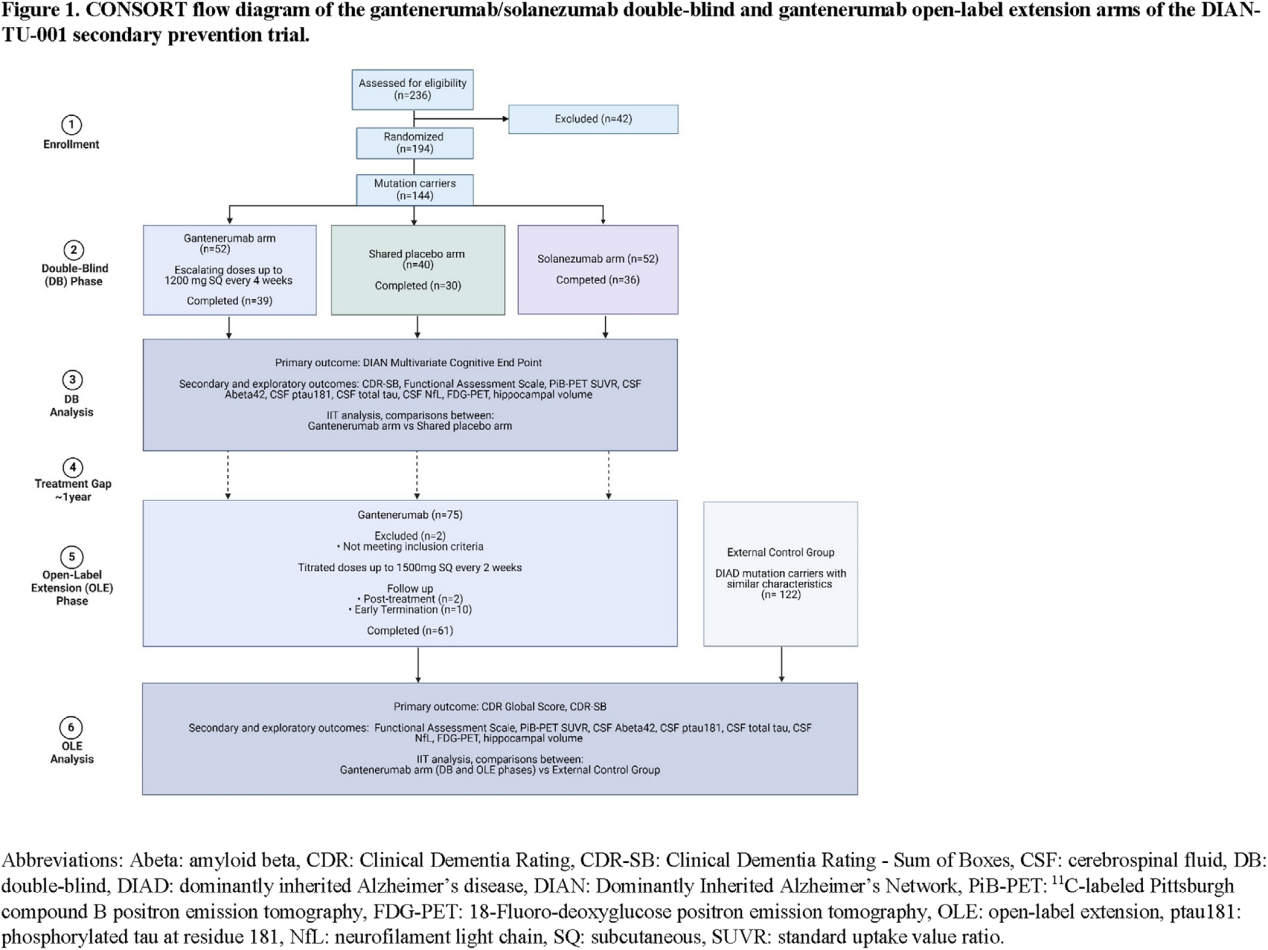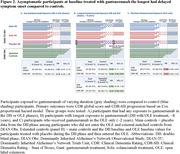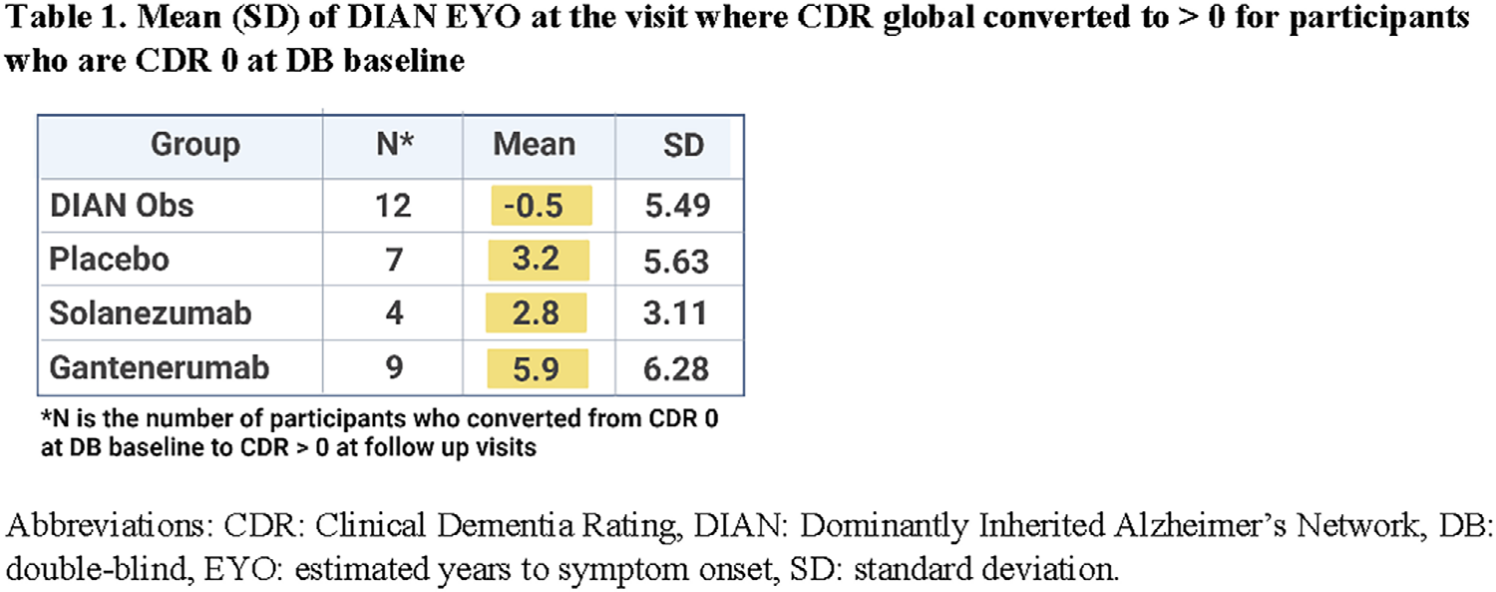# Delaying symptom onset in Dominantly Inherited Alzheimer’s Disease: Long‐term gantenerumab treatment results from the DIAN‐TU trial

**DOI:** 10.1002/alz.094602

**Published:** 2025-01-09

**Authors:** Randall J. Bateman, Yan Li, Eric McDade, Jorge J. Llibre‐Guerra, David B. Clifford, Alireza Atri, Susan Mills, Anna Santacruz, Guoqiao Wang, Charlene B Supnet, Tammie L.S. Benzinger, Brian A. Gordon, Laura Ibanez, Gregory Klein, Monika Baudler, Rachelle S. Doody, Geoffrey A. Kerchner

**Affiliations:** ^1^ Washington University in St. Louis, St. Louis, MO USA; ^2^ Knight Alzheimer Disease Research Center, St. Louis, MO USA; ^3^ Washington University in St. Louis, School of Medicine, St. Louis, MO USA; ^4^ Banner Health, Phoenix, AZ USA; ^5^ Washington University in St. Louis School of Medicine, St. Louis, MO USA; ^6^ Mallinckrodt Institute of Radiology, Washington University in St. Louis, St. Louis, MO USA; ^7^ F. Hoffmann‐La Roche Ltd, Basel Switzerland

## Abstract

**Background:**

Amyloid‐plaque removal by monoclonal antibody therapies slows progression in symptomatic Alzheimer’s disease (AD), but effects on preventing the onset of symptoms and dementia in asymptomatic people with amyloid plaques are unknown. We report the final primary and secondary outcomes of the Dominantly Inherited Alzheimer Network Trials Unit (DIAN‐TU) trial to evaluate amyloid‐plaque removal in delaying disease progression, including symptom onset, in symptomatic and asymptomatic dominantly inherited Alzheimer’s disease (DIAD) individuals treated for up to a decade.

**Method:**

This double‐blind, phase 2/3 trial (2012‐2019), followed by open‐label extension (OLE), investigated varying gantenerumab doses up to 1500 mg subcutaneous q2 weeks [NCT01760005]. The primary outcome was assessed for time to symptom onset and progression with the Cox proportional hazards model, comparing the treatment cohort to controls (double‐blind placebo participants who didn’t enter OLE, plus external controls). Estimated years to symptom onset (EYO) was included as a covariate and the average year of symptom onset relative to EYO was calculated. Other clinical and cognitive measures will be reported per the statistical analysis plan.

**Result:**

In asymptomatic participants at baseline treated with gantenerumab (n = 53), the overall HR (95% CI) was 1.00 (0.47, 2.14) for time to first Clinical Dementia Rating (CDR) global progression, and 0.76 (0.45, 1.29) for recurrent CDR‐Sum of Boxes (CDR‐SB) progression. In the longest‐treated group (n = 22, average 8 years), the HR (95% CI) was 0.50 (0.17, 1.47) for CDR global and 0.50 (0.25, 0.99) for CDR‐SB. The average age of dementia symptom onset for the longest gantenerumab exposure was 3 to 6 years later when compared to placebo or external controls.

**Conclusion:**

While no clinical effect was observed in the total group, results suggest potential delayed symptom onset and 50% dementia progression reduction in asymptomatic DIAD mutation carriers following long‐term, high‐dose gantenerumab treatment. Exploratory evaluation indicates that years of dementia free survival may be achieved with prevention. However, this analysis included a small cohort in an OLE with external controls, limiting conclusions. Ongoing follow‐up of this cohort will continue to inform future prevention trial designs and these results may represent the first clinical evidence of AD prevention with amyloid removal.